# Evidence of universal conformal invariance in living biological matter

**DOI:** 10.1038/s41567-025-02791-2

**Published:** 2025-03-14

**Authors:** Benjamin H. Andersen, Francisco M. R. Safara, Valeriia Grudtsyna, Oliver J. Meacock, Simon G. Andersen, William M. Durham, Nuno A. M. Araujo, Amin Doostmohammadi

**Affiliations:** 1 Niels Bohr Institute, University of Copenhagen, Copenhagen, Denmark; 2 Departamento de Física, Faculdade de Ciências, Universidade de Lisboa, Lisboa, Portugal; 3 Centro de Física Teórica e Computacional, Faculdade de Ciências, Universidade de Lisboa, Lisboa, Portugal; 4 Department of Fundamental Microbiology, University of Lausanne, Lausanne, Switzerland; 5 School of Mathematical and Physical Sciences, University of Sheffield, Sheffield, UK

**Keywords:** Fluid dynamics, Biological physics, Phase transitions and critical phenomena

## Abstract

The emergent dynamics of collective cellular movement are typically thought to depend on how cells interact with one another and the mechanisms used to drive motility, both of which exhibit remarkable diversity across different biological systems. Here we report experimental evidence of a universal feature in the patterns of flow that spontaneously emerge in groups of collectively moving cells. Specifically, we demonstrate that the flows generated by collectively moving dog kidney cells, human breast cancer cells and two different strains of pathogenic bacteria exhibit robust conformal invariance. We also show that the precise form of invariance in all four systems is described by the Schramm–Loewner evolution—a family of planar curves defined by a single parameter—and belongs to the percolation universality class. The presence of universal conformal invariance reveals that the macroscopic features of living biological matter exhibit universal translational, rotational and scale symmetries that are independent of the microscopic properties of its constituents. Our results show that flow patterns generated by different systems are highly conserved and that biological systems can be used to experimentally test predictions from the theories for conformally invariant structures.

## Abstract (web summary)

The flow features of cell monolayers depend on cellular interactions. Now four different types of cell monolayer are shown to exhibit robust conformal invariance that belongs to the percolation universality class.

## Main

Understanding the collective movement of large populations, and how it arises from its constituents, is a central problem in biology, ecology, materials science and physics^1–4^. In these living systems, work is produced at the level of an individual constituent, and this ‘activity’ is translated into patterns of collective motion at larger length scales through interactions between them^1,5^. However, many of the processes involved in collective movement, including the mechanisms that individual constituents use to propel themselves, the processes that give rise to interactions and the behavioural responses to stimuli, are incredibly diverse in different biological systems and are often difficult to decode^6,7^. Although many different models have been proposed to reproduce the specific pattern of collective movement made by particular organisms, we lack a general unifying theory or set of principles that unite the collective movement observed across distinct biological systems.

In contrast, the study of complex interactions between the components that make up certain inanimate materials, like metals and alloys, has led to the discovery of universal behaviour near the so-called critical regimes. In these conditions, the global macroscopic properties no longer depend on the specific properties of the individual constituents, but rather exhibit ‘universal’ behaviour^8^. The principles that give rise to this universality in inanimate materials have been described using the framework of conformal field theory^9,10^, which predicts how shapes and angles of structures are locally conserved across different systems, but not necessarily their length scales or curvatures. Although the techniques used to describe conformally invariant structures have long been used to make theoretical predictions in statistical mechanics and condensed-matter physics^9,10^ and to establish the universality of critical phenomena (for example, using numerical studies of turbulence^11–13^ and rigidity percolation^14,15^), the direct experimental observation of conformal invariance and robust universal critical behaviour in living matter remains elusive.

In this paper, we experimentally demonstrate that the patterns of collective movement observed in different types of living matter exhibit universal characteristics that transcend the particular properties of the cells from which they are composed. We show that vastly different systems, including colonies of pathogenic bacteria, groups of collectively moving dog kidney cells and human breast cancer cells, spontaneously generate flows that exhibit a universal conformal invariance that can be described by the percolation universality class. This finding suggests that collective cellular movement, which plays an important role in many biological systems^3,16,17^, could potentially serve as a fundamental test bed for theories that are based on conformal symmetry.

**Fig. 1:**
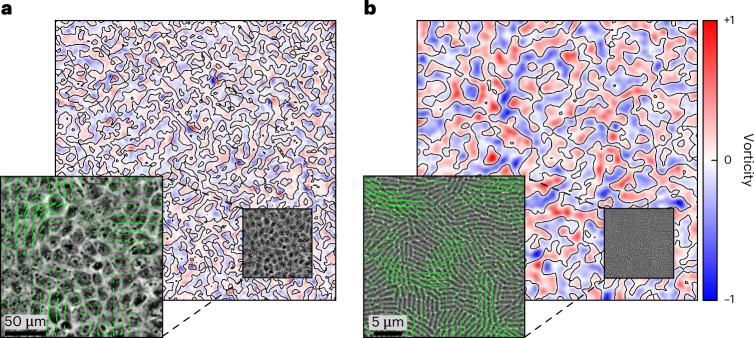
High-resolution measurements of the coherent flows from collectives of eukaryotic and prokaryotic cells. **a**,**b**, Representative velocity and vorticity fields observed in monolayers of eukaryotic MDCK cells (**a**) and prokaryotic WT *P. aeruginosa* cells (**b**). The colour map shows the local vorticity and the zero-vorticity contours are marked with black lines. The vorticity is normalized by its maximum value. The insets show a subset of cells within a single field of view, which have been overlaid with green arrows showing the local velocity. Here we have quantified movement using single-cell tracking (PIV), but we have also verified our results using PTV (Methods and Table 2).

We made high-resolution measurements of monolayers composed of four different cellular genotypes, including both prokaryotes and eukaryotes, to resolve whether we could identify common features in their collective motility. For prokaryotes, we studied the opportunistic pathogen *Pseudomonas aeruginosa*, which uses tiny grappling hooks called pili to pull itself along solid surfaces, a process known as twitching motility^18^. We considered two different strains of this rod-shaped bacteria—wild-type (WT) PAO1 and a deletion mutant *ΔpilH* lacking one of the response regulators in the Pil-Chp system, which causes it to become hyperpiliated, move faster and form longer cells than its parental WT^18–20^. For the eukaryotic cells, we considered the commonly studied Madin–Darby canine kidney (MDCK) cells^21^ and aggressive human breast cancer cells (MCF-7)^22^. Each of these genotypes forms monolayers through in situ growth. Although complex three-dimensional structures can emerge at later times^18^, all of the systems studied here exhibit two-dimensional (2D) collective patterns of motion. Vortical flow structures, a characteristic feature of the disordered flows observed in wide diversity of different systems^23^, are observed in all four of the cellular genotypes investigated here (Fig. 1 shows examples). Each vortex exhibits either clockwise or counterclockwise rotation, and the line that sits at the boundary between flows that rotate in opposite directions—the zero-vorticity contour—provides a measure of the underlying structure of the flow.

**Fig. 2:**
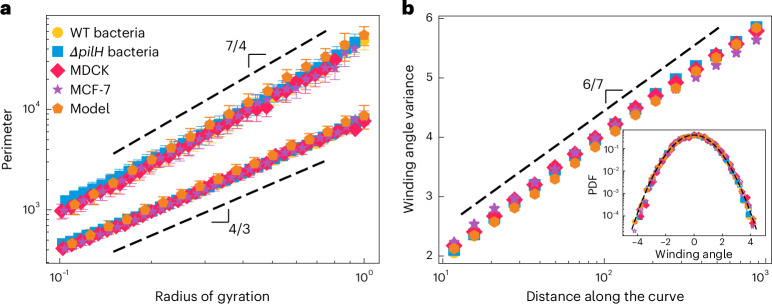
Vorticity contours from four distinct cellular systems exhibit the same patterns of scale and conformal invariance, which is recapitulated using a continuum model of an active fluid. **a**, Perimeter of contours as a function of their radius of gyration for two prokaryotic and two eukaryotic genotypes, including WT *P. aeruginosa* bacteria (yellow circles) and a hyperpilated *ΔpilH* *P. aeruginosa* mutant (blue squares) that individually move faster, and MDCK cells (red diamonds) and MCF-7 human breast cancer cells (purple stars). Here we separately analysed the complete perimeter and accessible external perimeter of the contours (Extended Data Fig. 3). We found that the experimental data for all four genotypes collapsed onto lines with slopes of approximately 7/4 and 4/3 for the two different perimeter measurements. The flow fields produced by a numerical model of active fluids (Methods) generated vorticity contours with a power-law dependency in close agreement with that observed in the experiments. The perimeter and radius of gyration is normalized by the radius of gyration of the largest vorticity cluster in their respective systems *R*_g,max_ (Methods). **b**, Variance in the distribution of the winding angle, plotted here as a function of distance along the curve for the four experimental systems and numerical model, all of which exhibit the same logarithmic scaling with a slope of 6/7 (dashed line). The inset shows the distribution of winding angles for a fixed distance along the contour, which is closely approximated by a Gaussian (dashed line). Both findings are consistent with that predicted for conformally invariant curves, which exhibit the same fractal dimension that we obtained for our data in **a**. The dashed lines correspond to a slope of 6/7 and a standard Gaussian distribution. The inset also shows that the winding angles are obtained for segments of contours with lengths of 64 (filled symbols) and 512 (empty symbols), and measured relative to the average angle of the contour. Here we show the mean (symbol) and s.d. (error bar) from *n* > 85 separate measurements of the flow field for each dataset (Methods). Source data

To compare how the flow structure varies across the four different experimental systems, we first measure the fractal dimension of the vorticity contours by plotting the perimeter of closed contours as a function of their radius of gyration. Surprisingly, without any fitting, special scaling or free parameters, the results for all four different experiments collapse on the same line and exhibit the same power-law behaviour (Fig. 2a). This provides concrete evidence of scale invariance and indicates that the flows generated by these diverse cellular systems share the same generic features. Interestingly, the slope of the perimeter–gyration radius plot, or fractal dimension, is *D* = 7/4 for the complete perimeter and D_*_ = 4/3 for the accessible external perimeter (Extended Data Fig. 3), and satisfies the duality relation 4(*D* – 1)(D_*_ – 1) = 1, conjectured for conformally invariant curves^24^. This finding suggests that these biological flow structures, in addition to being scale invariant, could exhibit much richer conformal symmetries^25^.

To test whether our experimental data demonstrate conformal invariance, we calculated the winding angle of the vorticity contours across the four different experimental systems. The winding angle is defined as the angle between two points on a contour that are separated by a given distance measured along the contour (Methods). The winding angle measures how much a curve turns as one proceeds along its length, characterizing the rotational behaviour of the curve, which is critical to understand the geometry of fractal structures. In addition, the winding angle is used to test whether a curve is conformally invariant, which occurs when its statistical properties remain unchanged under conformal transformations. For conformally invariant curves, (1) the winding angles are Gaussian distributed and (2) the variance in the distribution of winding angles increases logarithmically with the length of the curve^26^. Our experimental data are in close agreement with both predictions for conformal invariance—with both metrics collapsing the data from the vorticity contours of the four cellular systems onto the same line (Fig. 2b and Extended Data Fig. 5). Moreover, the rate at which the variance of the winding angle increases with the logarithm of the length is predicted to scale as *α* = 2(*D* – 1)/*D* for conformally invariant curves^26^. Thus, for the fractal dimension of *D* = 7/4 that we measured in Fig. 2a, we would predict that *α* = 6/7, which is supported by our direct measurements of variance (Fig. 2b).

**Fig. 3:**
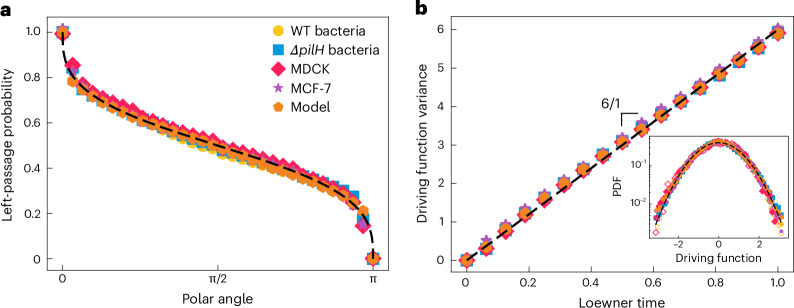
Resolving the underlying universality class of vorticity contours using two independent methods. **a**, Left-passage probability is defined as the probability that a point in space is on the right side of the contour for a given polar angle. Data from all four cellular genotypes and the results from the numerical model are in close agreement with Schramm’s formula for *κ* = 6 (dashed black line)^34^. **b**, Time dependence of the variance of the driving function obtained from a unique conformal slit map^33^. The dashed black line shows the result for one-dimensional Brownian motion with *κ* = 6. The inset shows the probability distribution of the driving function, rescaled by *κt*, where *t* is the Loewner time. Here the data at two different times (*t* = 0.25 and *t* = 0.75) are shown, which collapse onto the same curve (Methods). Source data

Our results strongly suggest that the flows spontaneously generated by diverse cellular genotypes exhibit robust conformal invariance, indicating that very different biological systems might be characterized by a common set of scaling laws. We next sought to ascertain if we could resolve which universality class these biological flows belong to. One of the central mathematical breakthroughs of the last few decades was to demonstrate that certain systems with conformal invariance and domain Markov property can be described, in the scaling limit of interfaces, by a family of planar curves defined by a single parameter *κ*. This formalism is known as the Schramm–Loewner evolution (SLE)^27,28^. The value of *κ* distinguishes different fundamental statistical mechanics models at criticality and, thus, resolves the universality class that a system belongs to^29–31^. To determine if the vorticity contours in the cellular systems are SLE curves, we extracted the *κ* parameter from the four experimental systems. We used two distinct and independent methods^32^: (1) directly calculating the driving function^33^ and (2) measuring the left-passage probability, comparing both to the analytic predictions for the SLE^34^ (Methods). The driving function captures the diffusivity of the curve in the SLE process. Physically, it represents how the curve evolves and changes direction and helps in understanding the underlying stochastic processes. The left-passage probability measures how likely it is for the curve to pass to the left of a given point as one proceeds further along the curve, which provides insights into the spatial distribution and geometry of the curve. This is crucial for understanding phenomena in which the connectivity and clustering of components are key. These two measurements, thus, provide independent ways of assessing if a curve can be described as an SLE curve and to determine the diffusivity parameter *κ* of the underlying SLE process. Although the left-passage probability indirectly measures the diffusivity of the curve, the driving function directly measures *κ*. For all the four cellular genotypes in our experiments, both methods consistently yielded *κ* = 6 (Fig. 3). The value of *κ* = 6 is also consistent with the estimated fractal dimension *D* (Fig. 2a), which, for SLE, is related to *κ* as *D* = 1 + *κ*/8 (ref. ^35^). This particular value of *κ* has an important physical meaning, as it has been uniquely proven that for *κ* = 6, the SLE curves correspond to the contours of critical percolation clusters and have the locality property (such that the properties only depend on the immediate neighbourhood)^30,36^. As such, our analyses reveal that the vorticity contours found in the four different cellular systems are not only conformally invariant but they also fall into the universality class of percolation.

Our experimental results indicate that diverse cellular types collectively generate flows with remarkably similar patterns of scale and conformal invariance, implying that the physical mechanisms that underlie the flow structures are highly conserved. Although many different physical models of active matter have been developed to approximate specific types of cell and the processes unique to them^2,5,37^, we tested whether a generic model could recapitulate our experimental observations. We used a simple continuum model in which a nematic order parameter (corresponding to cell orientation) was coupled to an incompressible velocity field (Methods). The two main parameters are activity *ζ* (represented by active stress generation in the velocity equation) and elasticity (represented by the elastic constant *K* that penalizes deformations). Dimensional analysis of the governing equations yields a characteristic length scale of √(*K/ζ)*, which defines the fundamental length scale of the flow. We find that the vorticity contours of this minimal model recapitulate each of the measurements observed in our experimental systems, including the fractal dimension *D* = 7/4 (Fig. 2a), winding angle scaling *α* = 6/7 (Fig. 2b) and the scaling of the driving function and left-passage probability *κ* = 6 (Fig. 3 and Table 1). Since elastic distortions in nematic materials can be screened at lengths comparable to the thickness of the nematic layer, we further explored the limit of small elasticity by varying the orientational elasticity in the model. The results show that for the same activity level, each of the estimates of the fractal dimension, winding angle scaling and scaling of the driving function change by less than 1% when we reduced the orientational elasticity (*K*) in our model by a factor of 500. This is because the isolines span much larger distances than the active length scale that is controlled by *K*. Although differences in cell morphology, intercellular adhesion, mechanotransduction and the mechanisms that give rise to local alignment can affect the patterns of collective motility^7,38,39^, the results of our continuum model imply that such idiosyncratic characteristics do not materially influence the scale and conformally invariant flow patterns, but rather are a generic feature of collective cellular flows.

**Table 1:** Measurements of four different cellular genotypes and numerical simulations

			Continuum model	Experimental data				Percolation universality class
				Bacterial cells		Eukaryotic cells		
				WT	*ΔpilH*	MDCK	MCF-7	
**Scale invariance**	Fractal dimension	*D*	1.75 ± 0.01	1.72 ± 0.02	1.72 ± 0.04	1.74 ± 0.02	1.74 ± 0.03	7/4 = 1.75
**Conformal invariance**	Winding angle	*α*	0.853 ± 0.005	0.86 ± 0.01	0.87 ± 0.01	0.85 ± 0.01	0.87 ± 0.02	6/7 ≈ 0.857
**SLE**	Left-passage probability	*κ*	6.02 ± 0.02	5.97 ± 0.05	5.96 ± 0.05	5.95 ± 0.03	5.95 ± 0.06	6
	Driving function	*κ*	5.96 ± 0.05	6.03 ± 0.06	5.96 ± 0.06	5.98 ± 0.04	5.93 ± 0.04	6

The values are calculated from the velocity fields obtained from PTV (Methods) and the errors represent the standard deviation about the mean.

The observed scaling of the vorticity contours from both experiments and model are compatible with SLE with *κ* = 6, for more than two decades in range. This was confirmed using two independent methods (Fig. 3 and Table 1). Remarkably, this finding demonstrates that although the collective cellular motility spontaneously generates patterns of flow with length scales much larger than that of individual cells (Fig. 1)^3,38^ and, thus, exhibit long-range order^40–49^, the associated vorticity contours are local and fall into the same universality class as those from random percolation^30^. Additionally, cell monolayers and bacterial colonies can exhibit patterns of coherent translational motion, giant density fluctuations and cells can even undergo volume fluctuations^50–52^. However, the observed conformal invariance of the vorticity contours are expected to be robust to such translation, dilation or shrinkage, characterized by finite drift or divergence in velocity fields. This is because both translation and dilation/shrinkage are angle-preserving conformal transformations, and conformal invariance comprises translational, rotational and scale invariance.

Moreover, the collective cellular motion we studied here is driven far from equilibrium by the motility of individual cells that continuously inject energy into the system at small scales. The observation of conformal invariance in collective cellular flows that are continuously driven far from thermodynamic equilibrium presents both challenges and new opportunities for the development of non-equilibrium conformal field theories^53^. It is important to note that our observations of conformal invariance were conducted on 2D monolayers of cells and the output of 2D simulations, so that the contours of vorticity form planar curves. Indeed, the predictions of SLE only apply to curves in two dimensions and there is, so far, no formal extension to surfaces in three dimensions. Nevertheless, the extension of these ideas to three-dimensional active matter flows would be a fascinating area to explore in future work.

These results suggest that the theories used to describe conformally invariant structures might have a much broader range of applications than previously anticipated. SLE-6 is a hallmark of conformal invariance and scaling behaviour, and is typically observed in systems at critical points, such as the percolation and phase transitions observed in models from statistical mechanics. Although our results do not necessarily indicate that the collective movement we observe is operating at the critical point of a phase transition, many different biological systems are thought to be poised near these critical points in their respective parameter space^54,55^. This so-called criticality is hypothesized to endow biological systems with flexibility by allowing them to easily switch between regimes that exhibit qualitatively different behaviours. Our findings, thus, indicate that the rigorous mathematical framework developed to study conformally invariant structures could potentially lead to new methods to detect and understand critical phenomena in biology.

Although collective movement is observed in diverse biological systems, that observed in microscopic cellular systems is particularly amenable to experimental analysis because in situ cell division rapidly gives rise to large genetically identical populations, the 2D movement of monolayers of cells can be readily imaged and the environmental conditions can be carefully controlled. Similar to the collective cellular motility studied here, many different living systems are formed of strongly interacting components driven far from thermal equilibrium and exhibit complex vortical patterns, including subcellular flows^56,57^, synthetic active material^1,58,59^, animal swarms^60,61^ and in vitro reconstitutions of cytoskeletal transport systems^62–64^. In addition, emergent vortical structures also shape many important processes in biology including cell differentiation^16^, cartilage regeneration^65^, embryogenesis^66^, signalling waves that propagate along cell membranes^67^ and between cells^68^, vortical waves associated with cardiac arrhythmia^69^ and spiral-like patterns of brain activity linked to cognitive processing^70^. We speculate that such biological processes might not only serve as a novel test bed to validate predictions based on conformal symmetry but this robust symmetry might also lead to the development of new analytical techniques to identify the fundamental mechanisms that give rise to both function and dysfunction in complex biological systems.

## Methods

### Experimental protocols

#### Eukaryotes

**Cell culture and preparation of monolayer assay**

MDCK-II cells stably expressing E-cadherin:red fluorescent protein were cultured in Dulbecco’s modified Eagle’s medium (low glucose, GlutaMAX supplement, pyruvate) supplemented with 10% fetal bovine serum (Gibco), 100 U ml^–1^ penicillin–streptomycin (Gibco), 1,000 μg ml^–1^ sodium bicarbonate and 500 μg ml^–1^ G-418 (Roche). MCF-7 cells were cultured in Dulbecco’s modified Eagle’s medium (high glucose, pyruvate; Gibco) supplemented with 10% fetal bovine serum (Gibco) and 100 U ml^–1^ penicillin–streptomycin (Gibco). Both cell lines were tested to ensure they did not contain mycoplasma and both were cultured at 37 °C with 5% CO_2_.

Cells were imaged in eight-well glass-bottom μ-slides (ibidi) that were pretreated with 10 μg ml^–1^ fibronectin human plasma in phosphate-buffered saline (pH 7.4; Gibco) and incubated for 30 min at 37 °C before cell seeding. Cells were seeded onto the surface at a density of approximately 2,000 cells mm^–2^ for MDCK cells and 3,000 cells mm^–2^ for MCF-7 cells, and were incubated for approximately 24 h to form a monolayer before imaging. For each eukaryotic cell type we imaged 8 different monolayers, collecting 12 different images from each.

**Live-cell imaging**

Cells were stained with Hoechst 33342 (Thermo Scientific) using a concentration of 3 μg ml^–1^ for MDCK cells and 6 μg ml^–1^ for MCF-7 cells in phosphate-buffered saline at 37 °C for 5 min. Cells were then washed three times with phosphate-buffered saline and immersed in fresh media before imaging. Samples were imaged using a Nikon ECLIPSE Ti microscope (running NIS Elements v.4.13.03) equipped with a H201-K-FRAME Okolab chamber, heating system (Okolab) and a CO_2_ pump (Okolab), which maintained them at 37 °C and at 5% CO_2_. The nuclei of MDCK and MCF-7 cells were imaged using wide-field microscopy for approximately 2–4 h with a 15 min interval between subsequent frames, using an Andor Neo 5.5 scientific complementary metal–oxide–semiconductor camera, ×4 Plan Fluor objective and Lumencor SOLA light engine. The time series were *x*–*y*-drift corrected using the Fast4DReg plug-in^71,72^ in Fiji. Fluorescent images of the nuclei were preprocessed using the smoothing, contrast enhancement and background subtraction tools in Fiji, in that order.

**Image analysis**

Particle image velocimetry (PIV) of eukaryotic monolayers was performed using PIVlab^73^. Spurious velocity vectors were identified and replaced via interpolation using PIVlab’s built-in in tools and then the velocity fields were smoothed using the ‘smooth’ function in MATLAB^74^. We then used interpolation to generate a set of vectors with a spacing of 10 μm.

For the particle-tracking velocimetry (PTV) analyses, we segmented and tracked individual cell nuclei in the time-lapse images using the Python module CellSegmentationTracker (https://github.com/simonguld/CellSegmentationTracker), which utilizes both Cellpose^75^ and TrackMate^76^. We used the pretrained deep learning model called ‘Nuclei’ in Cellpose to segment our images. The resulting Lagrangian cell trajectories were coarse grained onto a Eulerian grid with a spacing of 10 μm. Data points at the image boundaries were cropped to avoid edge artefacts.

Although we observe that our PIV and PTV measurements are highly correlated with one another, a perfect one-to-one agreement is not observed (Extended Data Fig. 2). However, our measurements of the fractal dimension, winding angle and SLE diffusivity are remarkably robust to the method used to quantify collective cell movement (Table 2).

**Table 2:** Estimates calculated using PTV and PIV measurements of the velocity fields are remarkably similar to one another

			Experimental data			
			Bacteria		Eukaryotic cell	
			WT	*ΔpilH*	MDCK	MCF-7
**Scale invariance**	Fractal dimension	*D*	0%	0%	0%	0%
**Conformal invariance**	Winding angle	*α*	1.1%	0.0%	1.2%	2.2%
**SLE**	Left-passage probability	*κ*	0.5%	0.3%	1.6%	0.8%
	Driving function	*κ*	2.5%	1.6%	1.0%	0.3%

The numbers reported here are the percentage difference between the parameters estimated using PTV data and PIV data.

#### Prokaryotes

**Cell culture and preparation of monolayer assay**

The WT *P. aeruginosa* and the corresponding hyperpilated *ΔpilH* mutant used here were previously published and characterized^18,20^. We streaked –80 °C freezer stocks onto 1.5% (w/v) Luria broth (LB) agar plates and incubated them overnight at 37 °C. Single colonies were picked and then used to inoculate the shaken liquid cultures that were then incubated overnight in liquid LB at 37 °C. The next day, overnight cultures were diluted 30-fold in fresh LB and returned to the 37 °C incubator, resulting in exponential phase cells after 2 h. Immediately before being used in the colony experiments, the optical density at 600 nm (OD_600_) of these cultures was adjusted to 0.05 using fresh LB. We then spotted 1 μl of the resulting culture onto a 0.8% LB agar pad and inverted it into a glass-bottom Petri dish (175 μm glass thickness, MatTek), as previously described^18^. The resulting subsurface colonies were then incubated overnight on the bench to allow them to develop a confluent monolayer at the edge of the subsurface colony. All assays were conducted at room temperature. The LB medium used here was composed of 10 g l^–1^ tryptone (Bacto brand, BD), 5 g l^–1^ NaCl (Fisher Scientific) and 5 g l^–1^ yeast extract (Bacto brand, BD).

**Live-cell imaging**

Time series of bacterial motility were captured using bright-field microscopy with a Nikon Ti-E inverted microscope outfitted with a Perfect Focus System, a Plan Apochromat ×100 objective, a Hamamatsu Flash 4.0 v2 camera and NIS-Elements software (v.4.51.01). We used the ×1.5 zoom feature on the microscope’s body, which increased the overall magnification to ×150. For each bacterial strain we collected 600 images of a single monolayer of cells at a rate of one frame per second. We then used PIV/PTV to measure cell velocities between subsequent images. We then subsampled these so that we processed every sixth velocity profile through our analysis pipelines.

**Image analysis**

PIV was performed on the bacterial data using a similar approach to the epithelial data. We used PIVlab^73^ to quantify the collective movement with a final vector spacing of ~1 μm. Occasionally (<2.5% of total), we observed very large spurious velocity vectors that were typically associated with regions with low cell density. These vectors were identified using a 0.75 μm s^−1^ velocity filter and were replaced using the PIVlab’s built-in interpolation tools.

PTV of bacterial data was performed using the feature-assisted segmenter/tracker (FAST)^77^, which enables the segmentation and tracking of individual bacteria within densely packed *P. aeruginosa* monolayers (further methodological details are provided elsewhere^18^). This yielded >100,000 cell trajectories for both WT and *ΔpilH* datasets. These were then coarse grained by overlaying a lattice on top of the imaged region and averaging the instantaneous movement vectors of all the cells within each lattice site, which was then repeated for each time step resulting in a time-varying flow field.

**Extended Data Fig. 1:**
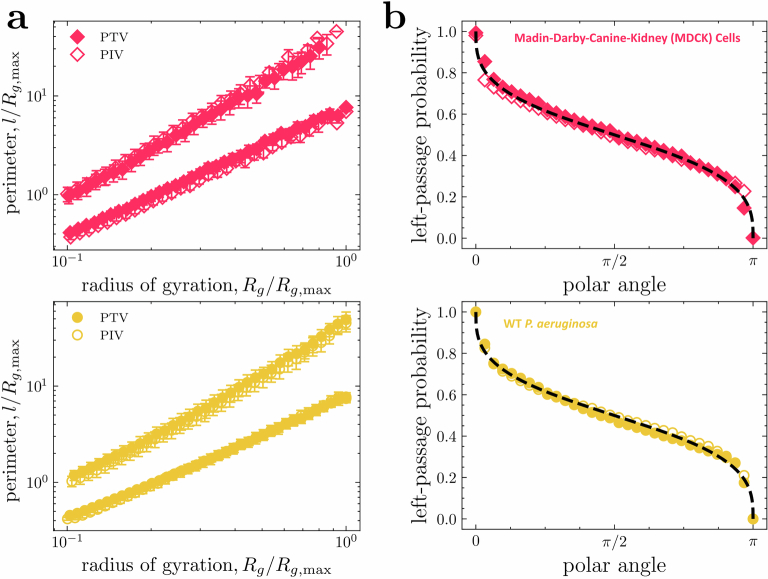
The method used to quantify collective cell movement does not appreciably affect our measurements of scale and conformal invariance. We measured the cell movement using both Particle Image Velocimetry (PIV, open symbols) and direct cell tracking (or Particle Tracking Velocimetry, PTV, filled symbols) and used each to calculate a respective set of zero-vorticity isocontours (see Materials and Methods for further details). **(a)** The complete perimeter and accessible external perimeter as a function of their radius of gyration using the isocontours obtained from the two different methods (analogous to that shown in Fig. 2a). Open symbols show PIV data, closed symbols show PTV data. **(b)** The left-passage probability as a function of the polar coordinate calculated using data from the two different methods, plotted alongside the analytic curve for *κ* = 6 (dashed black line). Top panels shows results for monolayers of MDCK cells and bottom panels shows results for wild-type *P*. *aeruginosa* cells. Error bars indicate the s.d. about the mean for the *n* > 85 flow field measurements that were obtained for each dataset. Source data

A comparison of the scale and conformal invariance measurements from PIV and PTV analyses is shown in Extended Data Fig. 1, demonstrating strong agreement between the two methods.

### Continuum model

We use a minimal, coarse-grained continuum model of suspended active nematogens that extends the Beris–Edwards equations^78^ for passive nematic liquid-crystal hydrodynamics, which are solved here using a hybrid lattice Boltzmann and finite difference method^79^. Relevant variables are the velocity field *u*_*i*_ as the slow variable, and the 2D, traceless and symmetric nematic order parameter *Q*_*ij*_ = 2*S*(*n*_*i*_*n*_*j*_ – *δ*_*ij*_/2). This second-rank tensor represents orientational order. The scalar order parameter *S* and director *n*_*i*_ are its largest eigenvalue and corresponding eigenvector, which encode the magnitude and direction of the nematic ordering, respectively. The governing dynamics consist of three coupled continuum equations describing an incompressible Stokes flow (at zero Reynolds number), which applies to cellular systems^80^, and the spatiotemporal evolution of the nematic order-parameter field, respectively:

1 $$0={\partial }_{j}{\Pi }_{ij},\quad {\partial }_{i}{u}_{i}=0$$

2 $$({\partial }_{t}+{u}_{k}{\partial }_{k}){Q}_{ij}-{S}_{ij}=\varGamma {H}_{ij}.$$

In equation (2), the generalized advection term *S*_*ij*_ = (*λE*_*ik*_ + *Ω*_*ik*_)(*Q*_*kj*_ + *δ*_*kj*_/2) + (*Q*_*ik*_ + *δ*_*ik*_/2)(*λE*_*kj*_ – *Ω*_*kj*_) – 2*λ*(*Q*_*ij*_ + *δ*_*ij*_/2)(*Q*_*kl*_∂_*k*_*u*_*l*_) is a co-rotational term, expressing the response of the nematic ordering to flow gradients (that is, any shear flow will either turn or tumble the nematogens) described by the strain rate tensor *E*_*ij*_ = (∂_*i*_*u*_*j*_ + ∂_*j*_*u*_*i*_)/2 and the vorticity tensor *Ω*_*ij*_ = (∂_*i*_*u*_*j*_ – ∂_*j*_*u*_*i*_)/2. The alignment parameter *λ* regulates whether this collective response of the nematogens to shear flow is dominated by strain or vorticity. The sign of *λ* denotes the shape of the nematogens, with *λ* > 0 and *λ* < 0 corresponding to a rod-like and disc-like shape, respectively. The molecular field $${H}_{ij}=-\delta {\mathcal{F}}/\delta {Q}_{ij}+({\delta }_{ij}/2){\rm{Tr}}\left(\delta {\mathcal{F}}/\delta {Q}_{kl}\right)$$ ensures that the nematic ordering relaxes diffusely to a minimum of the free energy $${\mathcal{F}}=K{\left({\partial }_{k}{Q}_{ij}\right)}^{2}$$ on a timescale set by the diffusive constant *Γ*, with *K* being the Frank elastic constant. The stress tensor *Π*_*ij*_ consists of three parts: viscous stress $${\Pi }_{ij}^{{\rm{viscous}}}=2\mu {E}_{ij}$$, elastic stress $${\Pi }_{ij}^{{\rm{elastic}}}=-P{\delta }_{ij}+2\lambda \left({Q}_{ij}+{\delta }_{ij}/2\right)\left({Q}_{kl}{H}_{lk}\right)$$$$-\lambda {H}_{ik}\left({Q}_{kj}+{\delta }_{kj}/2\right)-\lambda \left({Q}_{ik}+{\delta }_{ik}/2\right){H}_{kj}-$$$${\partial }_{i}{Q}_{kl}\left(\delta {\mathcal{F}}/\delta {\partial }_{j}{Q}_{lk}\right)+{Q}_{ik}{H}_{kj}-{H}_{ik}{Q}_{kj}$$ and active stress $${\Pi }_{ij}^{{\rm{active}}}=-\zeta {Q}_{ij}$$. Here *P* is the pressure and *ζ* sets both strength and nature of the activity, with *ζ* > 0 and *ζ* < 0 characterizing extensile and contractile nematogens, respectively. The elastic stress introduces backflow and the active stress implies that any gradient in the nematic ordering generates flows^81,82^.

The simulations shown here were conducted on a 2D square domain having dimensions of 4,096 × 4,096 with periodic boundary conditions. The lattice spacing and time step are taken as unity and the additional parameters are listed in the following table:

**Table:** 

Rotational diffusion, *Γ*	Elasticity, *K*	Viscosity, *μ*	Alignment, *λ*	Activity, *ζ*
0.05	0.05	1.0	1.0	0.1

Note that to keep the model as minimal as possible, no Landau–de Gennes bulk free energy is included in the definition of the free energy density, so that any potential local nematic ordering is solely induced by the activity. All of the model parameters are reported in lattice units.

### Statistical analyses of vortical flow structures

#### Calculating local vorticity and zero-vorticity isocontours

The vorticity field *ω* is obtained from the velocity field data ${({u}_{x},{u}_{y})}^{{\mathsf{T}}}$, using *ω* = ∂_*x*_*u*_*y*_ – ∂_*y*_*u*_*x*_. Both of the spatial derivatives are numerically computed using a five-point stencil at every grid point. To identify the locations of the zero-vorticity contours, we then calculated a binary field from the vorticity field using the ternary expression: 1 if *w* > 0, else 0. Contours of zero vorticity were then traced using a marching squares algorithm that preserves orientation, that is, always keeping sites of positive vorticity on its right (Extended Data Fig. 4).

#### Fractal dimension

We calculated the fractal dimension of vorticity clusters using both their complete and accessible external perimeters. Clusters are identified and labelled using a two-pass binary connected-component labelling algorithm^83^. The complete perimeter is identified by tracing the contour of a cluster according to the above contour-tracing algorithm. The corresponding accessible external boundary is constructed by dilating the boundary morphology and its perimeter is yet again measured using the contour-tracing algorithm (Extended Data Fig. 3).

The fractal dimension is measured by comparing the cluster perimeter *l* to its radius of gyration *R*_g_, for a large sample of vorticity clusters. The radius of gyration is computed as the positional mean square displacement from its centre of mass:

3 $${R}_{i}^{2}=\frac{1}{| {s}_{i}| }\sum _{n,m\in {s}_{i}}{({r}_{n}-{r}_{m})}^{2}\quad \,\text{and}\,\quad {R}_{g}^{2}=\frac{1}{N}\mathop{\sum }\limits_{i=1}^{N}{R}_{i}^{2},$$

where *s*_*i*_ denotes the set of lattice sites belonging to the *i*th cluster and ∣*s*_*i*_∣ is its cardinality. For the complete and external perimeters, we expect $l \sim {R}_{{\rm{g}}}^{{{D}}}$ and $l \sim {R}_{\rm{g}}^{{D}_{* }}$, respectively. For the zero-vorticity contours in our experiments and model, we find that the fractal dimension *D* of the complete perimeter is *D* = 1 + *κ*/8 = 7/4, for *κ* = 6, in agreement with the expected value for SLE_*κ*=6_ (ref. ^35^). By duality, the accessible external boundary is similarly conjectured to be SLE_8/3_ (ref. ^24^), implying that its fractal dimension D_*_ is related to *D* by the duality relation 4(*D* – 1)(D_*_ – 1) = 1 and consequently agrees with D_*_ = 4/3.

The perimeter and radius of gyration (Fig. 2a) have been normalized by the radius of gyration of the largest vorticity cluster in their respective systems, which are as follows:

**Table:** 

WT bacteria	*ΔpilH* bacteria	MDCK	MCF-7	Model
193 μm	200 μm	1,150 μm	1,130 μm	$828\sqrt{K/\zeta }$

Here the scaling factor used for the model is reported in units of active length scale $\sqrt{K/\zeta }$.

#### SLE contours

To detect the candidate SLE contours, we follow the procedure used elsewhere^11^ to study zero-vorticity isolines in the reverse cascade of classical 2D turbulence using data generated using a numerical model of the Navier–Stokes equations. To this end and in line with previous studies^12,84^, we used chordal SLE because it is mathematically less complex and more straightforward to calculate compared with radial or dipolar SLE. Chordal SLE describes curves that start and end at fixed boundary points of the upper-half plane and can be used to characterize a number of physical quantities, such as the interfaces in critical percolation and boundaries of clusters in statistical mechanics. Radial SLE describes curves from a boundary point to an interior point, and dipolar SLE involves curves between two boundary points with additional force points, making them more complex. Candidate chordal SLE_*κ*_ traces are identified using the following procedure (Extended Data Fig. 4).

1. Start with a binarized vorticity field, in which regions of positive vorticity are distinguished from negative vorticity (see the ‘Calculating local vorticity and zero-vorticity isocontours’ section).
2. In the complex plane, draw a horizontal line representing the real axis across the binary vorticity field;
3. The origin is defined at the intersection of a zero-vorticity contour and the real axis.
4. Consider an ‘explorer’ who starts at the origin and travels along the zero-vorticity contour such that regions of positive vorticity are always on the explorer’s right side.
5. If the explorer returns to the real axis, it should travel along that axis and preserve its previous orientation, until the explorer can re-enter the upper-half plane to again travel along the zero-vorticity contour with the region of positive vorticity on his right side.

This procedure faithfully reproduces the statistics of chordal SLE_*κ*_ in the scaling limit if and only if the isocontour satisfies the locality property of SLE_6_ (ref. ^85^), meaning that it does not ‘feel’ like the boundary before reaching it.

#### Winding angle

The winding angle *θ*_*j*_ of a curve sampled at the points ${\{{z}_{j}\}}_{j = 0}^{l}$ is defined as the cumulative sum ${\theta }_{j}=\mathop{\sum }\nolimits_{i = 1}^{\,j}{\alpha }_{i}$ of the local turning angles *α*_*i*_. The turning angle *α*_*i*_ is given by the angle between the two consecutive line segments [*z*_*i*−1_, *z*_*i*_] and [*z*_*i*_, *z*_*i*+1_] (Extended Data Fig. 5a). For putative chordal SLE_*κ*_ traces, in the scaling limit, we expect that the winding angle at a given distance *s* along the trace is Gaussian distributed and that the variance grows logarithmically^26,86–88^ in accordance with

4 $${\rm{Var}}(\theta )=a+\frac{2\kappa }{8+\kappa }\log [s].$$

This expression is used to directly calculate the diffusivity (*κ*), where *a* is a constant.

#### Left-passage probability

The probability that a chordal SLE_*κ*_ trace, with *κ* ∈ [0, 8), passes to the left of the point *z* = *ρ*e^i*ϕ*^ in the upper-half plane depends only its argument *ϕ* and is given by Schramm’s formula^34^

5 $${P}_{\kappa }(\phi )=\frac{1}{2}+\frac{\varGamma (4/\kappa )}{\sqrt{\uppi }\varGamma \left(\frac{8-\kappa }{2\kappa }\right)}\cot {(\phi )}_{2}{F}_{1}\left[\frac{1}{2},\frac{4}{\kappa },\frac{3}{2},-{\cot }^{2}(\phi )\right],$$

where *Γ* is Euler’s gamma function and _2_*F*_1_ is Gauss’ hypergeometric function.

To measure this left-passage probability, we fixed a finite set ${\mathcal{S}}$ of points in the upper-half plane and measured the probability *P*(*z*) that a putative chordal SLE_*κ*_ trace passes to the left of these points. Following another work^89^, the diffusivity *κ* is estimated by minimizing the weighted mean square deviation (Extended Data Fig. 5b):

6 $$Q(\kappa )=\frac{N-1}{| {\mathcal{S}}| }\sum _{z\in {\mathcal{S}}}\frac{{\left[P(z)-{P}_{k}(\arg z)\right]}^{2}}{P(z)[1-P(z)]},$$

where *N* is the number of samples and $| {\mathcal{S}}|$ denotes the cardinality of the set ${\mathcal{S}}$.

#### Driving function

The stochastic driving function ${U}_{t}:[0,T\,]\to {\mathbb{R}}$ encoding the information of a chordal SLE_*κ*_ curve is measured by numerically integrating the forward chordal Loewner equation^90^:

7 $${\partial }_{t}{g}_{t}(z)=\frac{2}{{g}_{t}(z)-{U}_{t}},$$

with initial condition *g*_0_(*z*) = *z*. The numerical integration scheme is simple: we introduced a partition 0 = *t*_0_ < *t*_1_ < … < *t*_*n*_ = *T* for time interval [0, *T*] and approximated the driving function ${U}_{{t}_{k}}={\delta }_{k}$ as constant on each short time interval *Δ*_*k*_ = *t*_*k*_ – *t*_*k*−1_. Then, the conformal map ${g}_{{t}_{k}}(z)$ was obtained by explicitly solving the Loewner equation (7). Although there are many such solutions^91^, in this study, we arguably used the most simple one—the vertical slit map^33^:

8 $${g}_{{t}_{k}}(z)=\sqrt{{(z-{\delta }_{k})}^{2}+4{\Delta }_{k}}+{\delta }_{k},$$

which simply projects the vertical slit extending from *δ*_*k*_ to ${\delta }_{k}+2{\rm{i}}\sqrt{{\Delta }_{k}}$ onto the real axis (Extended Data Fig. 6a). Presume we have a putative chordal SLE_*κ*_ trace sampled at the points $\{{z}_{0}^{0}=0,{z}_{1}^{0},\ldots ,{z}_{l}^{0}\}$, the Loewner times *t*_*k*_ and driving function ${U}_{{t}_{k}}$ are computed iteratively by the successive application of a vertical slit map (8). At each iteration step, the points $\{{z}_{k}^{k-1},{z}_{k+1}^{k-1},\ldots ,{z}_{l}^{k-1}\}$ get mapped onto the reduced sequence of points $\{{z}_{k+1}^{k}={g}_{{t}_{k}}({z}_{k+1}^{k-1}),\ldots ,{z}_{l}^{k}={g}_{{t}_{k}}({z}_{l}^{k-1})\}$ (Extended Data Fig. 6b).

To claim that zero-vorticity curves truly are chordal SLE_*κ*_ curves, the extracted driving function ${U}_{{t}_{k}}$ must be a Brownian process. However, in addition to its variance scaling linearly with the Loewner time according to Var(*U*_*t*_) = *κt*, it should be Gaussian distributed at every time instance, too. However, as demonstrated earlier^92^, these are not a sufficient test on their own, as these criteria can also be satisfied by non-SLE_*κ*_ processes. Following another work^89^, studying the autocorrelation function

9 $$C(t;\tau )=\frac{{\rm{Cov}}(\delta {U}_{t+\tau },\delta {U}_{t})}{\sqrt{{\rm{Var}}(\delta {U}_{t+\tau }){\rm{Var}}(\delta {U}_{t})}}$$

of the driving function increments *δU*_*t*_ confirms that the driving function is a Markovian process (Extended Data Fig. 7).

### Reporting summary

Further information on research design is available in the Nature Portfolio Reporting Summary linked to this article.

## Online content

Any methods, additional references, Nature Portfolio reporting summaries, source data, extended data, supplementary information, acknowledgements, peer review information; details of author contributions and competing interests; and statements of data and code availability are available at 10.1038/s41567-025-02791-2.

## Supplementary information

Reporting Summary

## Source data

Source Data Fig. 2 Source data for Fig. 2a,b and Fig. 2b (inset).

Source Data Fig. 3 Source data for Fig. 3a,b and Fig. 3b (inset).

Source Data Extended Data Fig. 1 Source data for Extended Data Fig. 1a,b.

**Extended Data Fig. 2:**
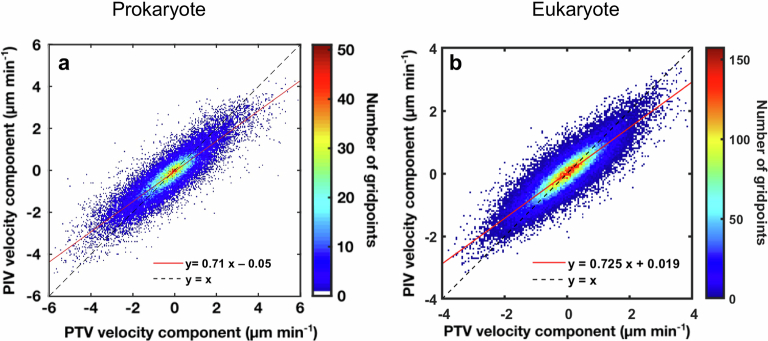
Comparison between velocity field obtained from tracking (PTV) and particle image velocimetry (PIV). Shown here are the covariant plots for (**a**) WT bacteria and (**b**) MDCK monolayer. The datapoints would fall along a line with a slope of 1 (dashed black line) if these two measurements had precisely the same value. Source data

**Extended Data Fig. 3:**
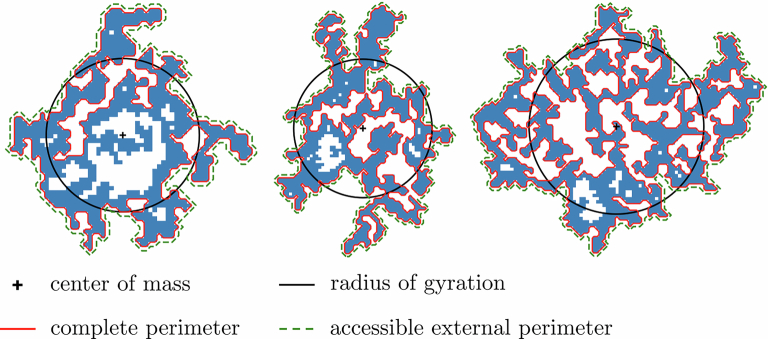
Three representative clusters of positive vorticity to illustrate the methods used in our analyses of contour perimeter and gyration radius. Each of these clusters of positive vorticity (shown in blue) was detected in a monolayer of wild-type *P*. *aeruginosa* using velocity fields obtained by PTV (Materials and Methods). Solid red lines show the complete perimeter, dashed green lines show the accessible external perimeter, black circles show the radius of gyration, and black crosses show the center of mass of each cluster.

**Extended Data Fig. 4:**
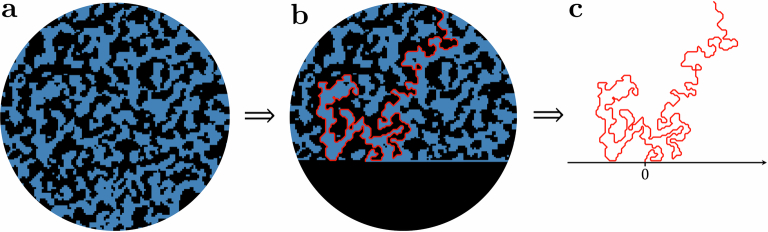
The procedure used to calculate candidate chordal SLE_*κ*_ traces used in our analyses. **(a)** Blue/black sites show regions of positive/negative vorticity, measured in monolayer of wild-type *P*. *aeruginosa* using PTV (Materials and Methods). **(b)** The intersection of zero-vorticity contour (shown in red) with a horizontal line. **(c)** The labeled zero-vorticity contour is mapped to the upper half-plane.

**Extended Data Fig. 5:**
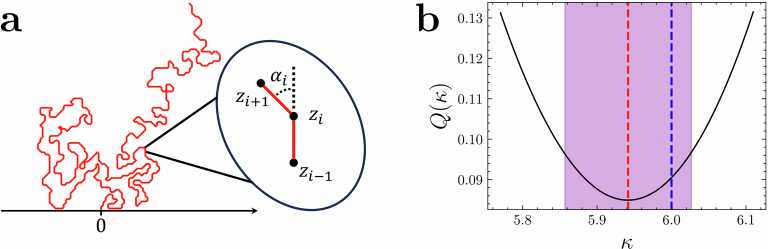
The procedure used to calculate the winding angle. **(a)** The turning angle, *α*_*i*_, is defined as angle between the two consecutive line segments [*z*_*i*−1_, *z*_*i*_] and [*z*_*i*_, *z*_*i*+1_]. **(b)** Weighted mean square deviation *Q*(*κ*) as function of the diffusivity *κ* for WT *P*. *aeruginosa* PTV data. The blue vertical dashed line is a guide to eye at *κ* = 6. The minimum mean square deviation occurs at *κ* = 5.94 ± 0.08 (red vertical dashed line). The shaded magenta region corresponds to the 95% confidence interval about the minimum.

**Extended Data Fig. 6:**
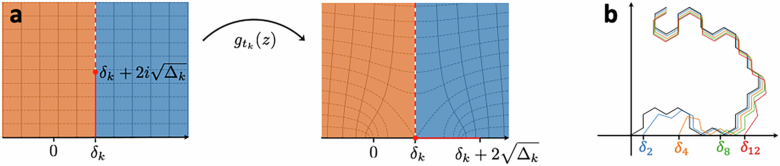
Calculating the driving function using a vertical slip-map. **(a)** Schematic of the vertical slit-map Eq. (8). **(b)** The vertical zipper algorithm: the original path (black) is conformally mapped onto the real axis.

Source Data Extended Data Fig. 2 Source data for Extended Data Fig. 2a,b.

**Extended Data Fig. 7:**
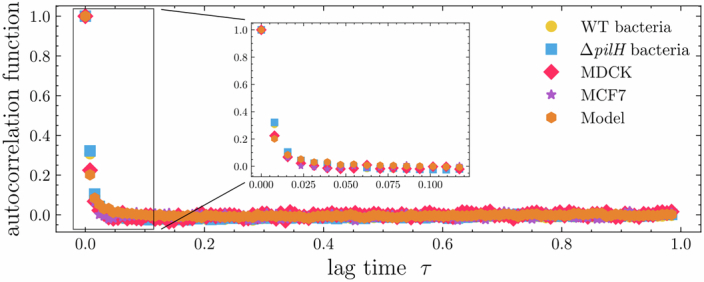
The autocorrelation of the driving function decays rapidly with lag time. This finding is consistent with the Markov property expected for Schramm-Loewner evolution contours in the scaling limit. The initial decay is due to the finite lattice spacing, which introduces short range correlations. Source data

Source Data Extended Data Fig. 7 Source data for Extended Data Fig. 7.

## Data Availability

The data that support the findings of this study are available at https://sid.erda.dk/cgi-sid/ls.py?share_id=enaibOX0oR. Source data are provided with this paper.

## Code availability

The CellSegmentationTracker package for analyses of the experimental data can be accessed via GitHub at https://github.com/simonguld/CellSegmentationTracker. The code for the numerical simulation can be accessed via GitLab at https://gitlab.nbi.ku.dk/active-intelligent-matter/mass-nematic. The feature-assisted segmenter/tracker (FAST) package can be accessed via Zenodo at 10.5281/zenodo.3630641 (ref. ^93^), with extensive documentation on its use and functionality available at https://mackdurham.group.shef.ac.uk/FAST_DokuWiki/dokuwiki.
